# New Physico-Chemical Analysis of Magnesium-Doped Hydroxyapatite in Dextran Matrix Nanocomposites

**DOI:** 10.3390/polym16010125

**Published:** 2023-12-29

**Authors:** Daniela Predoi, Steluta Carmen Ciobanu, Simona Liliana Iconaru, Ştefan Ţălu, Liliana Ghegoiu, Robert Saraiva Matos, Henrique Duarte da Fonseca Filho, Roxana Trusca

**Affiliations:** 1 National Institute of Materials Physics, Atomistilor Street, No. 405A, P.O. Box MG 07, 077125 Magurele, Romania; simonaiconaru@gmail.com (S.L.I.); ghegoiuliliana@gmail.com (L.G.); 2 The Directorate of Research, Development and Innovation Management (DMCDI), Technical University of Cluj-Napoca, 15 Constantin Daicoviciu St., 400020 Cluj-Napoca, Romania; stefan.talu@auto.utcluj.ro; 3 Amazonian Materials Group, Physics Department, Federal University of Amapá (UNIFAP), Macapá 68903-419, Amapá, Brazil; robert_fisic@unifap.br; 4 Laboratory of Synthesis of Nanomaterials and Nanoscopy (LSNN), Physics Department, Federal University of Amazonas-UFAM, Manaus 69067-005, Amazonas, Brazil; hdffilho@ufam.edu.br; 5 National Centre for Micro and Nanomaterials, University Politehnica of Bucharest, 060042 Bucharest, Romania; truscaroxana@yahoo.com

**Keywords:** biomedical applications, dextran, fractal features, hydroxyapatite, magnesium

## Abstract

The new magnesium-doped hydroxyapatite in dextran matrix (10MgHApD) nanocomposites were synthesized using coprecipitation technique. A spherical morphology was observed by scanning electron microscopy (SEM). The X-ray diffraction (XRD) characterization results show hydroxyapatite hexagonal phase formation. The element map scanning during the EDS analysis revealed homogenous distribution of constituent elements of calcium, phosphor, oxygen and magnesium. The presence of dextran in the sample was revealed by Fourier transform infrared (FTIR) spectroscopy. The antimicrobial activity of the 10MgHAPD nanocomposites was assessed by in vitro assays using *Staphylococcus aureus* ATCC 25923, *Pseudomonas aeruginosa* ATCC 27853, *Streptococcus mutans* ATCC 25175, *Porphyromonas gingivalis* ATCC 33277 and *Candida albicans* ATCC 10231 microbial strains. The results of the antimicrobial assays highlighted that the 10MgHApD nanocomposites presented excellent antimicrobial activity against all the tested microorganisms and for all the tested time intervals. Furthermore, the biocompatibility assays determined that the 10MgHApD nanocomposites did not exhibit any toxicity towards Human gingival fibroblast (HGF-1) cells.

## 1. Introduction

Dental caries is an expensive public health problem affecting up to 91% of adults (worldwide). The dental plaque formation usually occurs due to the microbial colonization of oral cavity surfaces [1]. Diet, age, oral hygiene routine, systemic and immune status are important factors that influence the apparition and development of dental plaque [2,3,4]. The excessive presence of sugar in the daily diet favors the apparition of dental caries [5]. 

Moreover, a diet rich in sugar favors the development of pathogens in the oral cavity (such as *Streptococcus mutans*), which leads to the formation of acidic and adherent biofilms that are difficult to combat. At the same time, these biofilms lead to a demineralization of dental enamel, thus favoring the apparition of caries [6]. Due to the nanotechnology progress in the medical field [7], materials that have in their composition zinc oxide, silver or magnesium ions were proposed as antibiofilm agents [8,9].

Dextran is a natural polysaccharide, and its use on humans is approved by FDA [10]. The efficiency of dextran-coated iron oxide nanozymes against oral biofilm development, together with their biocompatibility, was shown by Pratap et al. [11]. Moreover, the possibility of using calcium phosphate such as hydroxyapatite (Hap) as a dental filler was evaluated [12]. Studies show that the addition of Hap to dental composite leads to the reinforcement of cement mechanical properties [13]. HAp structure has the ability to allow a large number of substitutions with various ions, including magnesium, zinc, silver, etc. [14,15,16,17,18]. The addition of magnesium ions to the Hap structure is important due to its metabolic role in bone regeneration [19]. The Mg deficiency induces serious health problems, such as osteopenia/osteoporosis, because the lack of Mg in the body disturbs the activity of osteoblast cells [19,20]. For the development of biomaterials based on magnesium-doped hydroxyapatite, various synthesis routes were proposed: hydrothermal [21], precipitation [22], sol–gel [23], mechanochemical–hydrothermal [24], wet chemical [25], and microwave [26,27]. These approaches allow us to obtain nanomaterials with desired properties, such as morphology, dimension, biological properties, etc.

Polymeric nanoparticles have been used in many biomedical applications, such as drug delivery, tissue engineering, dentistry and imaging [28]. Usually, polymeric nanoparticles can be developed using various natural and/or synthetic polymers (e.g., polyethylene glycol (PEG), polylactic acid (PLA), poly(lactic-co-glycolic acid) (PLGA) and/or gelatin, alginate, albumin, chitosan, dextran etc.) [28]. Previous studies showed that polymeric nanoparticles exhibit a great potential for uses in drug delivery applications due to their biocompatibility and stability. Furthermore, polymeric nanoparticles were used for drug delivery and imaging applications due to their unique properties, such as high surface area to volume ratio and tunable size [29]. For example, in the work conducted by El-Meliegy et al. [30], the synthesis of a novel composite scaffold based on hydroxyapatite in dextran/chitosan polymeric matrix was reported. Their results highlighted that the presence of Hap nanoparticles in the polymeric matrix enhances the physicochemical properties of the obtained composite scaffolds [30]. In this context, Hap and dextran have been used for the development of polymeric nanoparticles with potential uses in various biomedical applications, such as drug delivery, tissue engineering, dentistry and imaging [31].

Shoba et al., in their work entitled “3D nano-bilayered spatially and functionally graded scaffold impregnated bromelain conjugated magnesium-doped hydroxyapatite nanoparticle for periodontal regeneration” revealed that scaffolds containing magnesium-doped hydroxyapatite possess an improved antibacterial activity and biocompatibility proving that such materials can be promising candidates for uses in periodontal tissue regeneration [32]. Moreover, in our previous antimicrobial study conducted on magnesium-doped hydroxyapatite suspension obtained by an adapted coprecipitation method, we underlined the efficacy of this biomaterial against *P. aeruginosa, S. aureus,* and *C. albicans* strains [33]. Also, we noticed that the antimicrobial activity of magnesium-doped hydroxyapatite is strongly correlated with the Mg concentration found in the samples. Therefore, a more efficient antimicrobial activity against Gram-positive strains (*B. subtilis* and *S. aureus*) was noticed in the case of an increased Mg concentration in the hydroxyapatite/chitosan composite samples when compared to those against Gram-negative strains [34]. Also, a study conducted on chitosan-coated magnesium-doped hydroxyapatite coatings highlighted the in vitro biocompatibility of the studied samples with the human fibroblast cell [34].

In the present study, we proposed the development of a novel biomaterial based on magnesium-doped hydroxyapatite in dextran matrix (10MgHApD). The research focused on the physicochemical characterization and antimicrobial evaluation of 10MgHApD nanocomposites with high potential to be applied in the dental field. 

## 2. Materials and Methods

### 2.1. Materials

The synthesis of magnesium-doped hydroxyapatite (Ca_10−x_Mg_x_(PO_4_)_6_(OH)_2_, x_Mg_ = 0.1 and [Ca + Mg]/P ratio equal with 1.67) in dextran matrix was conducted using ammonium hydrogen phosphate ((NH_4_)_2_HPO_4_), calcium nitrate tetrahydrate (Ca(NO_3_)_2_·4H_2_O), magnesium nitrate hexahydrate (Mg(NO_3_)_2_·6H_2_O) purchased from Sigma-Aldrich (St. Louis, MO, USA) with a purity of 99.97%. Dextran (H(C_6_H_10_O_5_)_n_, Mr ~ 40,000) was also purchased from Sigma-Aldrich (St. Louis, MO, USA). In the synthesis double-distilled water was used.

### 2.2. Synthesis of Magnesium-Doped Hydroxyapatite in Dextran Matrix Nanocomposites

Magnesium-doped hydroxyapatite in dextran (10MgHApD) matrix nanocomposites were obtained by the coprecipitation technique [35]. In order to achieve this purpose, the atomic ratio of (Ca + Mg)/P was 1.67. A solution (300 mL) containing (NH_4_)_2_∙HPO_4_ and 10% H(C_6_H_10_O_5_)_x_OH (10 g) was stirred for 30 min at 40 °C. in air. A similar procedure was followed for the solutions (300 mL) containing Ca(NO_3_)_2_∙4H_2_O and Mg(NO_3_)_2_∙6H_2_O. The solutions containing calcium and magnesium were dropped into the solution with dextran. The pH of synthesis was kept constant at 11 by adding NH_3_. After 5 h of stirring after the end of dripping, the resulting suspension was centrifuged and redispersed in 10% solution of dextran (10 g at 100 mL of double-distilled water). The procedure was rehearsed five times. The resulting precipitate after the last centrifugation was redispersed in a 10% dextran solution and stirred for 12 h at 60 °C in air. The final suspension was centrifugated, and the last precipitate was dried at 40 °C (in air) and called 10MgHApD and afterwards analyzed in the present study. 

### 2.3. Characterization Methods

#### 2.3.1. Scanning Electron Microscopy

A scanning electron microscope (FEI Quanta Inspect F, FEI Company, Hillsboro, Oregon, United States) equipped with an energy-dispersive X-ray (EDS) attachment was used to study the morphology of 10MgHApD nanocomposites. 

#### 2.3.2. X-ray Diffraction

X-ray diffraction (XRD) was used to examine the magnesium-doped hydroxyapatite (10MgHAp), 10MgHApD nanocomposites and dextran. The equipment for XRD analysis was a Bruker D8 Advance diffractometer with CuKα (λ = 1.5418 Å) radiation (Bruker, Karlsruhe, Germany), equipped with a high-efficiency LynxEye™ 1D linear detector. The patterns were achieved in the 2θ range 20–60°. The step size was 0.02° and the dwell time was 5 s. 

#### 2.3.3. Fourier Transform Infrared Spectroscopy

The presence of functional groups was established by Fourier transform infrared (FTIR) spectroscopy in attenuated total reflectance (ATR) mode. A Perkin Elmer Spectrum BX II spectrometer (Perkin Elmer, Waltham, MA, USA) equipped with a Pike-MIRacle ATR head with diamond-ZnSe crystal plate, having a diameter of 1.8 mm (Pike Technologies, Madison, WI, USA) was used. The spectra were acquired in the 450–3800 cm^−1^ spectral range. The resolution was 4 cm^−1^ and represented the average of 32 individual scans.

#### 2.3.4. Atomic Force Microscopy (AFM)

Detailed information regarding the morphology of the composites was achieved by atomic force microscopy (AFM) technique. For this purpose, the composite nanocomposites were pressed into pellets, and the surface topography of the pellet was studied using an instrument NT-MDT NTEGRA Probe Nano Laboratory (NT-MDT, Moscow, Russia). The measurements were performed at room temperature and in atmospheric conditions using semi-contact mode. Information about the morphology of the samples was obtained by recording AFM surface topographies on surface areas of 10 × 10, 5 × 5 and 3 × 3 µm^2^ using a silicon NT-MDT NSG01 cantilever (NT-MDT, Moscow, Russia) with a 35 nm gold layer. Information about the roughness of the samples was also obtained by calculating the roughness parameter *R_RMS_*. The recorded AFM data were processed using the 2.59 version of Gwyddion software (Department of Nanometrology, Czech Metrology Institute, Brno, Czech Republic) [36]. 

#### 2.3.5. Monofractal and Multifractal Analysis

Herein, the monofractal parameters were performed evaluate the surface microtexture spatial complexity. The fractal dimension (*FD*) was computed using the Mandelbrot box-counting method [37], and the Hurts coefficient was obtained by applying the formula *H* = (3 – *FD*) [37]. The Fractal succolarity (*F_S_*) was determined using Equation (1) [38], where *T*(*k*) represents the count of boxes of uniform sizes *T(n)*, *P_0_*(*T*(*k*)) denotes the occupation percentage within each box, *PR* represents the occupation pressure, and *p_c_* signifies the centroid’s position (*x,y*) representing the applied pressure on the corresponding box. This equation provides a quantifiable measure of *F_S_*, offering insights into the structural complexity and hole distribution across the analyzed surface [38].

(1) $$F_{s}\left(T\left(k\right),dir\right)=\frac{\sum_{k=1}^{n}P_{0}\left(T\left(k\right)\right)\cdot PR(T\left(k\right),p_{c})}{\sum_{k=1}^{n}PR(T\left(k\right),p_{c})}$$

The topographic entropy was computed using Shannon entropy [39], as defined in Equation (2) [40]. In this equation, the term $p_{ij}$ signifies the probability of pixels exhibiting discrepancies or not within the specified height range of the analyzed universe. The calculation of topographic entropy through Shannon entropy allows for a quantitative measure of the information content associated with the variability in pixel values across the analyzed topography [40].

(2) $$TE=-\sum_{i=1}^{N}\sum_{j=1}^{N}p_{ij}\cdot logp_{ij}$$

On the other hand, the multifractal theory is a mathematical framework employed to characterize intricate objects or systems showcasing substantial variations in their properties across various scales [38]. Its application is prevalent in the analysis of 3D spatial patterns, time series, and diverse complex phenomena. Serving as an extension of fractal theory, which centers on objects with self-similarity—patterns recurring at different scales—multifractal theory broadens this perspective to encapsulate the multifaceted variability observed in complex systems [41]. We use the partition function (Equation (3)) for expressing the mass exponent *(τ_q_)*, where $p_{i}\left(\varepsilon \right)=r_{i}(\varepsilon )/\sum_{k=1}^{N(\varepsilon )}r_{k}(\varepsilon )$ represents the probability of occupancy of the *i*-th cell within a resolution grid *ε*. The $r_{i}(\varepsilon )$ term denotes the cumulative fluctuation of the height around the mean value within the *i*-th square [37].

(3) $$Z(q,\varepsilon )=\sum_{i=1}^{N(\varepsilon )}p_{i}^{q}(\varepsilon )\sim \varepsilon^{\tau (q)}$$

The multifractal spectrum elucidates the variation in complexity associated with different exponents, such as Hölder exponent (α(q)), and this relationship is expressed by Equation (4) [37].

(4) $$f\left(\alpha \left(q\right)\right)=q\cdot \alpha \left(q\right)-\tau (q)$$

The mass exponent curve defines a relationship between moments of order (*q*) and generalized dimensions (*D_q_*) and can be expressed according to Equation (5) [37].

(5) $$D_{q}=\frac{\tau (q)}{\left(q-1\right)}$$

where $\alpha \left(q\right)=\frac{d\tau (q)}{dq}$ defines the connection between the ($\alpha \left(q\right)$) and the mass exponent (*τ*(*q*)) for a given value of *q*.

#### 2.3.6. In Vitro Antimicrobial Assays

The in vitro antimicrobial properties of 10MgHApD nanocomposites were studied with the aid of *Staphylococcus aureus* ATCC 25923, *Pseudomonas aeruginosa* ATCC 27853, *Streptococcus mutans* ATCC 25175, *Porphyromonas gingivalis* ATCC 33277 and *Candida albicans* ATCC 10231 microbial strains. The experiments were performed as previously described in [42]. For this purpose, 10MgHApD nanocomposites, as well as 10MgHAp and HAp nanoparticles, were exposed to 1.5 mL of microbial suspension of a standardized density equal to 5 × 10^6^ CFU/mL (colony forming units/mL). *S. aureus*, *P. aeruginosa* and *C. albicans* microbial suspensions of a density of approximately 5 × 10^6^ CFU/mL were prepared from 15 to 18 h. Solid cultures were grown in tryptone soy agar (TSA). Afterwards, the microbial suspensions were inoculated onto Muller Hinton agar (MHA) plates by swabbing. Afterwards, the suspensions were collected at different time intervals (24, 48 and 72 h) and incubated on Luria–Bertani (LB) agar medium for 24 h at 37 °C. *P. gingivalis* was grown on Brucella agar plates containing a blood agar base, yeast extract, glucose (4.5%) under anaerobic conditions (80% N_2_, 10% H_2_, 10% CO_2_). The colonies were harvested and resuspended in a Brain Heart Infusion (BHI) broth (Difco). *S. mutans* were cultured from single colonies in BHI (Difco) in an aerobic atmosphere with 5% CO_2_. The colonies were harvested and resuspended in BHI broth. The density of the microbial suspensions was adjusted by adding either *P. gingivalis* and *S. mutans* suspended in BHI broth or just pure BHI broth. The microbial suspensions were prepared as described above and then incubated for 24, 48 and 72 h, respectively with the 10MgHApD nanocomposites and 10MgHAp and HAp nanoparticles. As a positive control, a free microbial culture was assessed at the same time intervals. 

The microbial suspensions were prepared in phosphate-buffered saline (PBS) and then incubated for 24, 48 and 72 h, respectively, with the 10MgHApD nanocomposites. The values of the CFU/mL were determined. The experiments were performed in triplicate and the data were presented as mean ± standard deviation (SD). The statistical analysis was performed using the ANOVA single-factor test.

#### 2.3.7. In Vitro Biocompatibility Assay

The biocompatibility of the 10MgHApD nanocomposites was studied using a Human gingival fibroblasts (HGF-1) cell line. For this purpose, the cells were cultured using Dulbecco’s Modified Eagle’s Medium enriched with heat-inactivated fetal bovine at 37 °C in an atmosphere containing 95% air and 5% CO_2_. The HGF-1 cells were seeded in culture plates and were allowed to adhere for 24 h. Afterwards, the cultured cells were incubated with 10MgHApD nanocomposites for 24, 48 and 72 h. An untreated cell culture was used as control. The cell viability of the HGF-1 cells was determined with the aid of the reduction assay MTT [3-(4,5dimethylthiazolyl)-2,5-diphenyltetrazolium bromide]. To achieve this, the cells were seeded in 96-well plates (5 × 10^4^ cells/mL), incubated for 24 h, and then treated with the 10MgHApD nanocomposites. After 24, 48 and 72 h of incubation, the cells were washed using phosphate buffer saline (PBS) and incubated with 0.5 mg/mL MTT solution for 4 h. The HGF-1 cell viability was quantified by determining the optical density of the medium at 595 nm with the aid of a TECAN spectrophotometer. The percentage of the HGF-1 viable cells was quantified by rapport to the control sample, which was considered to have a viability of 100%.

## 3. Results and Discussion

### 3.1. X-ray Diffraction

**Figure 1:**
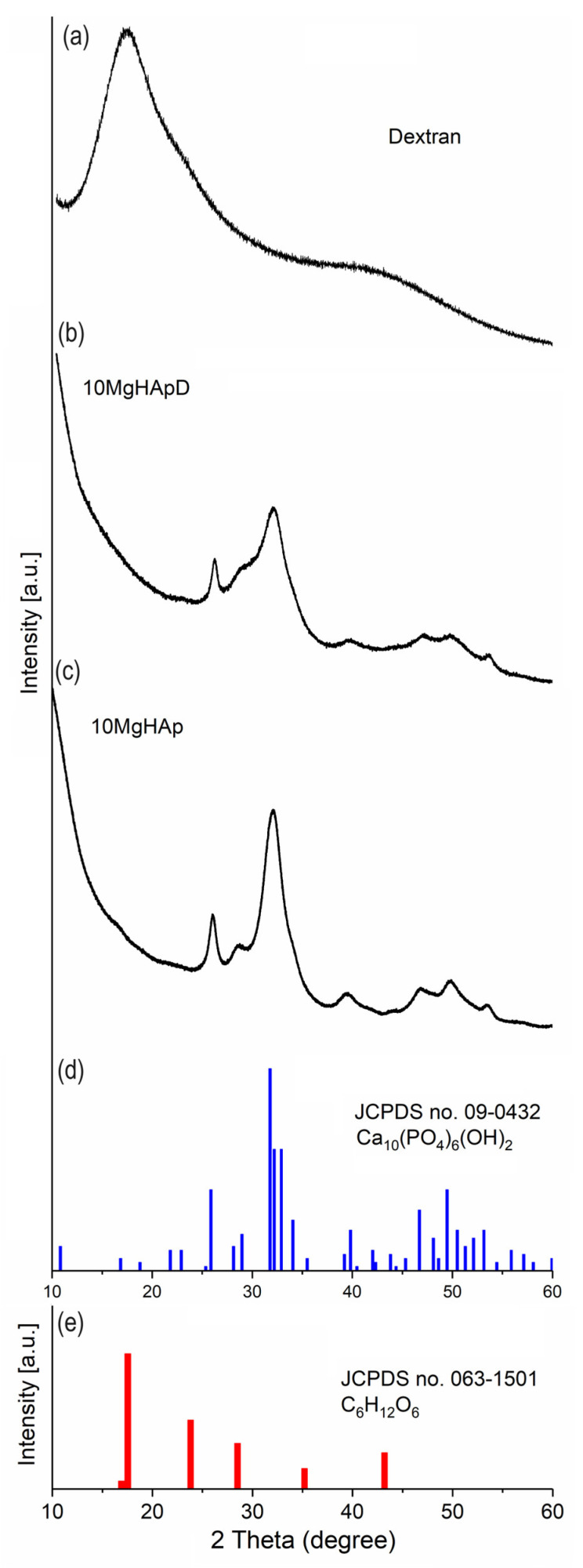
XRD spectrum of dextran (**a**), 10MgHApD nanocomposites (**b**) and 10MgHAp (**c**). The lines of the ICDD-PDF#9-432 reference file of hexagonal hydroxyapatite (**d**) and the ICDD-PDF#063-1501 reference file of dextran (**e**).

To assess the magnesium incorporated in hydroxyapatite coated with dextran, XRD studies were conducted. In Figure 1, XRD patterns of the dextran, magnesium-doped hydroxyapatite in dextran matrix (10MgHApD) and magnesium-doped hydroxyapatite (10MgHAp) nanocomposites are presented. The reference hexagonal patterns of hydroxyapatite (JCPDS no. 09-0432) and dextran (JCPDS no. 063-1501) are also shown.

The diffraction pattern of 10MgHAp and 10MgHApD was similar to that of the reference hexagonal HAp pattern (ICDD-PDF#09-432). The diffraction pattern of both samples highlights the fact that the particles have nanometric dimensions (20.1 ± 2 nm for 10MgHAp and 13.6 ± 4 nm in the case of the 10MgHApD sample). It is observed that the diffraction pattern of 10MgHApD shows wider peaks than in the case of the 10MgHAp sample [43]. This behavior could also be caused by the presence of dextran. Due to its presence, dextran can lead to a decrease in crystallinity [44].

### 3.2. Scanning Electron Microscopy

**Figure 2:**
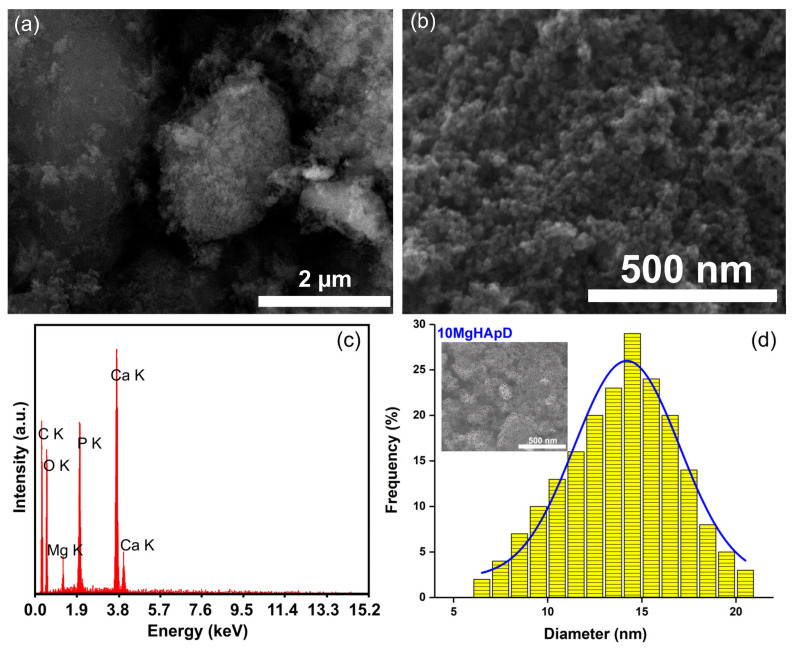
SEM micrograph at low (**a**) and high (**b**) resolution of 10MgHApD nanocomposites, EDS spectrum of 10MgHApD nanocomposites (**c**) and average particle size (**d**).

The morphology of the as-synthesized 10MgHApD nanocomposite material is shown in Figure 2. Figure 2a represents the SEM micrograph at low resolution, while Figure 2b shows the SEM micrograph of 10MgHApD nanocomposites at high resolution. The micrograph shown in Figure 2b exhibits nanometric particles with a spherical shape. The average particle size calculated after measuring approximately 500 particles was 14.5 ± 2 nm (Figure 2d). The inset of Figure 2d presents the micrograph on which approximately 500 particles are numbered. Typical EDS patterns establish six prominent peaks which confirm the presence of magnesium, calcium, phosphor, oxygen and carbon, respectively (Figure 2c).

**Figure 3:**
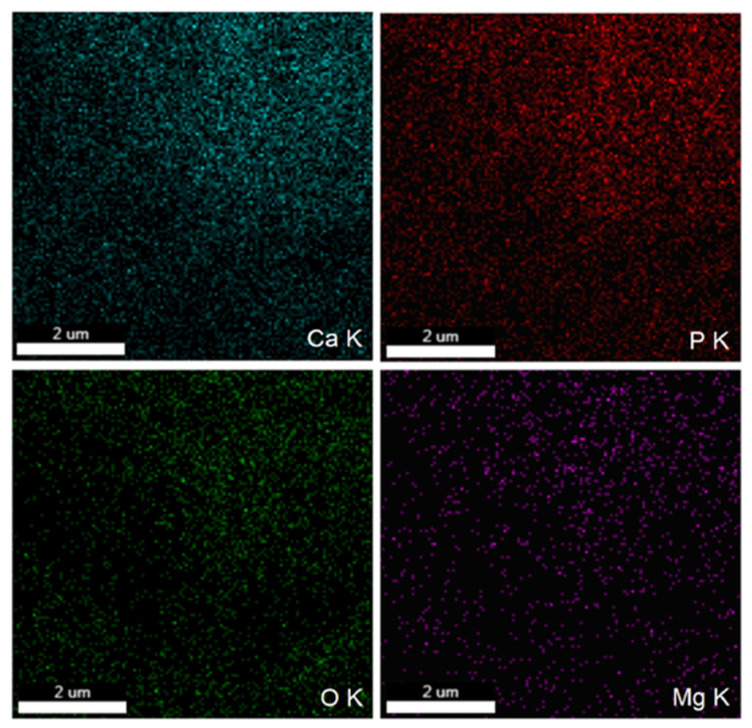
The EDS mapping of Ca, P, O and Mg in 10MgHApD nanocomposites.

The element map scanning during the EDS analysis was conducted from the region revealed in Figure 2a. The results regarding the element map scanning during the EDS analysis of 10MgHApD nanocomposites are exhibited in Figure 3. The homogenous distribution of constituent elements Ca, P, O and Mg is observed. The C element is not presented because it is not conclusive (the C contribution has two sources, the carbon band and the dextran from the synthesized sample).

### 3.3. Atomic Force Microscopy

**Figure 4:**
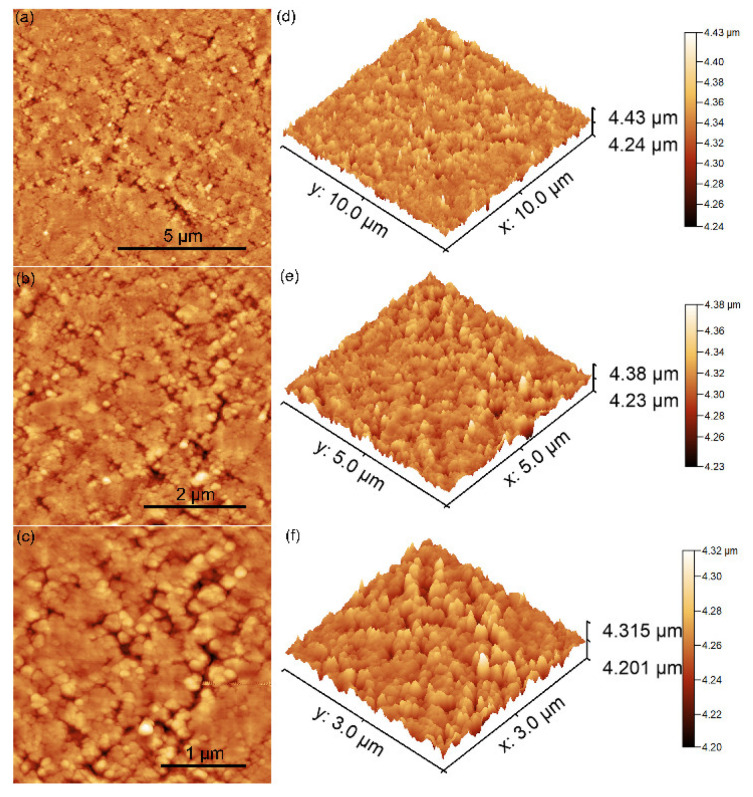
2D AFM images of 10MgHApD pellet’s topography recorded on an area of 10 × 10 µm^2^ (**a**), 5 × 5 µm^2^ (**b**), 3 × 3 µm^2^ (**c**) and their corresponding 3D representations (**d**–**f**).

The morphology of the 10MgHApD nanocomposites was further investigated using AFM technique. Information about the sample’s morphology was obtained by recording AFM topographies on surface areas of 10 × 10, 5 × 5 and 3 × 3 µm^2^ of the pellet surface topography obtained from the 10MgHApD nanocomposites nanocomposites. The results of the AFM studies are depicted in Figure 4a–f. 

The AFM topography reveals a surface with slight irregularities resulting from the process of obtaining the pallets. The homogeneous distribution of the agglomerates formed by nanoparticles was also observed. The AFM topography obtained on the surface area of 3 × 3 µm^2^ highlighted that the nanoparticles form agglomerates. In addition, the results suggested that the agglomeration of particles exhibited nanometric sizes. Both the 2D surface topography of the three investigated areas, as well as their 3D representation, emphasized that the nanocomposites present a uniform and homogenous morphology. Slight irregularities of the pellet surface could be observed. The roughness parameters, R_RMS_, calculated for the areas of 10 × 10, 5 × 5 and 3 × 3 µm^2^ of the pellet surface topography obtained from the 10MgHApD nanocomposites were 15.36, 13.56 and 10.91 nm, respectively. It can be seen that the obtained values are very close. The values of the roughness parameter suggest a homogeneous distribution of the agglomerates formed by nanoparticles on the surface of the pallets.

**Figure 5:**
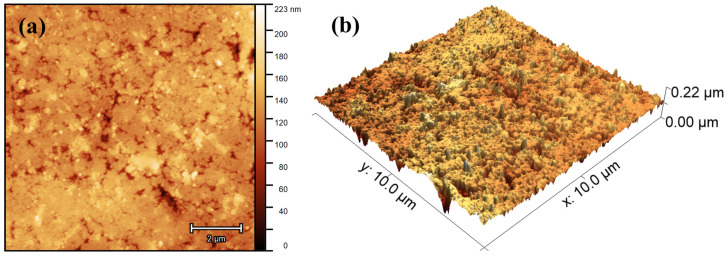
(**a**) 2D and (**b**) 3D representation of the atomic force microscopy (AFM) surface topography image of the 10MgHApD sample, with 10 × 10 μm scanned area.

The investigation of coating surfaces using AFM has become a cornerstone for assessing nanoscale topographic changes. With its capability to map both topography and mechanical properties, AFM plays a pivotal role in advancing nanotechnology, characterizing biomaterials, and optimizing devices [37]. In this regard, we assessed the morphology and microtexture of 10MgHApD nanocomposites across various scales. The comprehensive view of the overall morphology of the 10MgHApD pellet’s topography recorded on an area of 10 × 10 µm^2^ and its 3D spatial configurations is depicted in Figure 5. The 3D topographic map covering dimensions of 10 × 10 µm^2^ illustrates a relatively smooth surface with certain irregularities formed randomly along the surface following the process of obtaining the pallets. This behavior that appears on the surface of the pellets after the pressing process could be beneficial, contributing to the improvement of the adhesive properties of the surface [45] in order to develop applications in the biomedical field.

**Figure 6:**
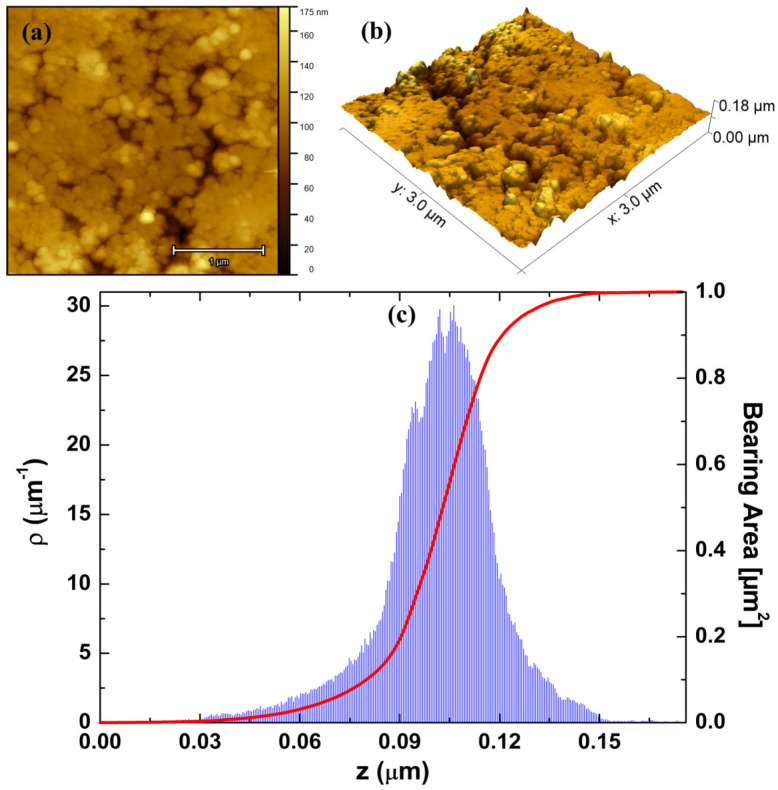
(**a**) 2D and (**b**) 3D AFM micrographs of 10MgHApD nanocomposites. (**c**) Height histogram and Abbot–Firestone curves from the respective image.

To gain deeper insights into the 3D spatial configuration of the vertical growth profile of the investigated surface, we conducted a detailed analysis of its microtexture. This involved utilizing 3D AFM topographic maps with dimensions of 3 × 3 µm^2^, as shown in Figure 6a,b. As it can be seen, the pellet surface appears nearly homogeneous and uniform, displaying a finely tuned vertical profile indicative of low topographic roughness. Additionally, the 10MgHApD particles are evenly distributed across the surface, showcasing sizes ranging from 50 to 200 nm. The average roughness (*Sa*) was computed to be 13 ± 0.2 nm, which is a markedly lower value than other values reported for different coatings, e.g., 980 nm [46] and 47 nm [47]. Such behavior was also observed for the other height ISO-based parameters: maximum peak height (*Sp*), maximum pit depth (*Sv*), and maximum height (*Sz*) (Table 1). Notably, the low roughness of the pellet surface suggests that the incorporation of MgHAp into the dextran matrix has a softening effect on the vertical profile of the investigated surface, with the particles being shaped and embedded by the polymer. Figure 6c shows the shape of the height distribution associated with the vertical profile of the pellet surface and its Abbot Firestone curve [39,48]. As observed, the height distribution of the investigated surface is centralized, a characteristic supported by the kurtosis value *Rku* ~3, signifying an almost perfectly platykurtic pattern [49]. Furthermore, the distribution is almost symmetric, which is supported by the skewness value *Rsk* ~0 [50] (Table 1). Additionally, the high quality of the investigated surface is also illustrated by the Abbot Firestone curve, characterized by its typical S shape. In this regard, the curve attains its peak value more rapidly at a specific relative height *z* value (in µm), providing evidence that the height distribution follows an almost normal behavior.

**Figure 7:**
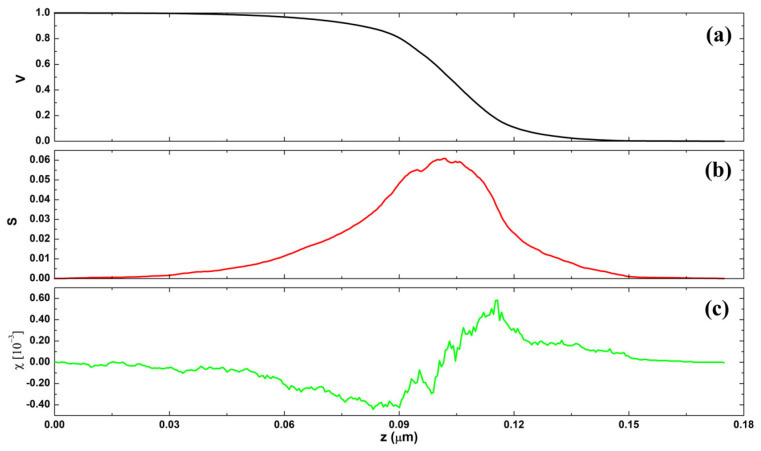
The MFs functionals of the surface of 10MgHApD obtained from AFM image for (**a**) Minkowski volume, (**b**) Minkowski boundary, and (**c**) Minkowski connectivity.

Minkowski Functionals (MFs) serve as geometric measurements employed to characterize and quantify the topological and morphological properties of geometric sets [51,52]. Their primary applications lie in morphological analysis, particularly in contexts such as image analysis and the study of porous materials [37]. The Minkowski volume (*V*) limit of the investigated surface, as depicted in Figure 7a, exhibits a characteristic S-like shape and approaches its minimum rapidly. This behavior indicates that the volume of material below a threshold is low, confirming the surface’s exceptionally smooth vertical profile. The Boundary Minkowski (*S*) (Figure 7b) depicts a higher maximum value in 0.06, with a distribution of points similar to a normal curve. This suggests that the surface boundary of the sample is intricate, containing distinctive features in its contour. Finally, the Minkowski connectivity (*χ*) exhibits a typical minimum negative and a sharp positive maximum value, as shown in Figure 7c. This suggests that the investigated surface has homogeneous surface percolation. This behavior may be associated with a regular distribution of gaps along the pellet surface. Notably, these aspects of the nanocomposite morphology align with the observations made about its morphological and microtextural properties (Figure 6a,b).

### 3.4. Monofractal Analysis

**Figure 8:**
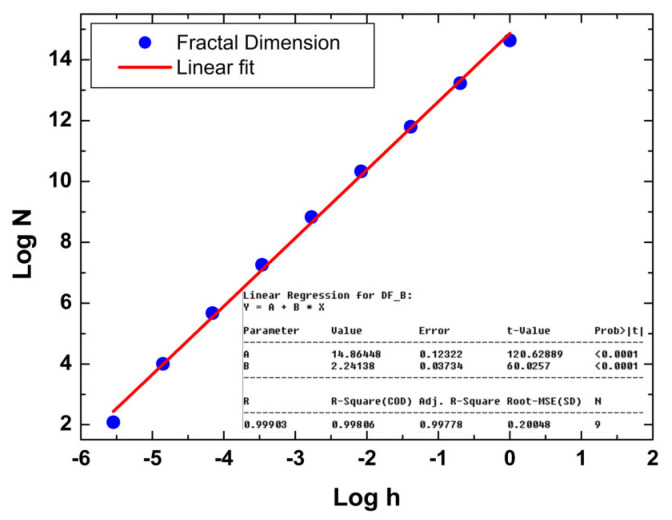
Representative fractal dimension determined using a cube counting method of 10MgHApD nanocomposites, obtained from AFM images shown in Figure 2.

Analyzing the spatial complexity of surfaces using monofractal mathematics is crucial for deciphering intricate 3D patterns of polymer surfaces in nanoscale [53]. This approach offers a profound understanding of shapes, facilitating the optimization of industrial processes, material design, and environmental modeling. By unraveling the underlying geometry, monofractal mathematics emerges as a valuable tool for enhancing efficiency and fostering innovation across various domains, e.g., biological [54], thin films [55], and biomedical [56]. In our monofractal approach, we employed the Mandelbrot box-counting method [37] to obtain the fractal dimension, whose fit is shown in Figure 8. Remarkably, the smoothness of the nanocomposite surface is associated with relatively low spatial complexity (*FD* < 2.5) (Table 2). Despite this, the surface exhibits an *FD* value > 2, indicating the presence of topographic irregularities that give rise to long-range correlations. The relatively low spatial complexity of the nanocomposites is also linked to the presence of low dominant spatial frequencies, as indicated by the high value of the Hurst coefficient (*H* > 0.5) (Table 2). Thus, the arrangement of the topographic heights in the pellet surface microtexture promotes the development of a surface with low roughness and 3D spatial complexity characterized by low spatial frequencies in the topographic profile.

Furthermore, we observed that the pellet surface is not highly porous (*FS* < 0.5), a characteristic that is not directly associated with the porous nature of the 10MgHApD structure. According to Ţălu et al. [38], a surface with ideal surface percolation has to display *FS* = 0.5, indicating a highly uniform hole distribution across the surface. In contrast, a more percolable surface is expected to exhibit *FS* > 0.5. In this regard, it is evident that the low roughness of the nanocomposites facilitated the development of a less porous surface, primarily attributed to the presence of dextran polymer in its structure. In addition, the average topographic entropy (*E*), a parameter linked to the uniformity of 3D spatial patterns in the distribution of topographic heights, was calculated to be 0.897 ± 0.006. This behavior implies the presence of more uniform spatial patterns (*E* < 0.5) than nonuniform ones (*E* > 0.5), indicating high surface quality and resistance of the coating. Moreover, surfaces with an *E* value ~1 tend to demonstrate homogeneous surface adhesion [40,57], which is advantageous for biological applications, including cell anchoring. In conclusion, our findings suggest that the 10MgHApD nanocomposites display monofractal behavior characterized by low spatial complexity, low surface percolation, and high topographic uniformity, largely attributed to the low surface roughness.

### 3.5. Multifractal Analysis

**Figure 9:**
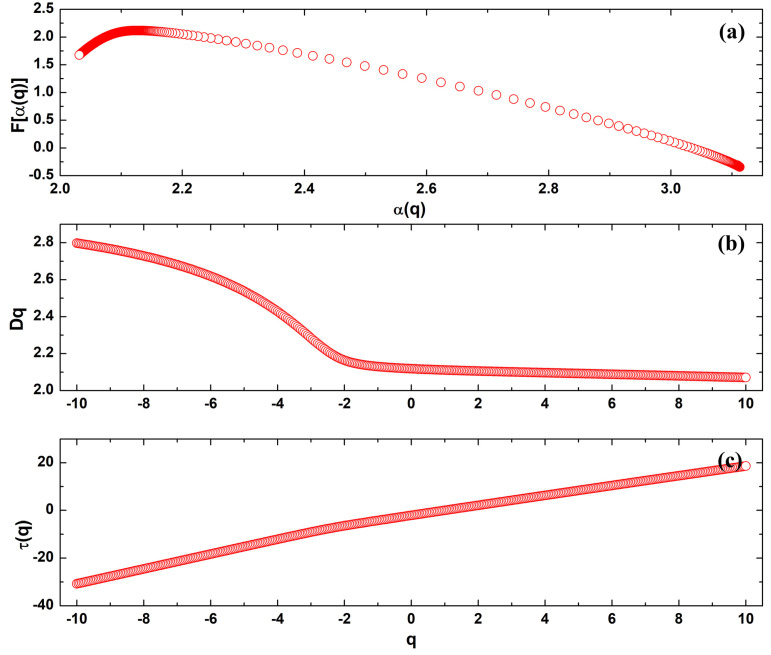
(**a**) Multifractal spectra (*f(α)* versus α), (**b**) generalized dimensions *D_q_*, and (**c**) mass exponent *τ(q)*, as a function of the order of moments computed for 10MgHApD nanocomposites obtained using their 3D AFM topographical map.

We conducted an in-depth examination of the pellet surface dynamics utilizing a multifractal methodology. In fact, it is recognized that monofractals have limitations as they solely rely on a single fractal dimension [37]. However, the analysis of multifractal behavior of a multifractal sample is crucial because it can address inhomogeneous surface complexity [37,57]. While the monofractal approach provides a global view of spatial complexity, multifractal analysis allows for a more refined description of local and regional variations using multiple scaling exponents [58,59]. This approach identifies specific scales of complexity, revealing nonlinear details and heterogeneities critical for full sample characterization, especially in systems where properties vary significantly at different spatial scales, e.g., roughness and surface isotropy. The analysis of 3D spatial patterns in the surface of 10MgHApD nanocomposites reveals unique multifractal properties, indicating significant structural complexity, as displayed in Figure 9. The multifractal parameters computed from the multifractal spectra of 10MgHApD nanocomposites are presented in Table 3. The multifractal spectra curve (Figure 9a), characterized by a downward concavity, serves as a hallmark of multifractal systems, with the maximum point corresponding to the Hausdorff dimension. This dimension offers insights into the three-dimensional complexity of the coating, highlighting structural variations at different scales. The spectra width (Δα = α_max_ − α_min_), calculated as 1.078, indicates a broad range of structural sizes within the coating. Higher values suggest substantial diversity in structural scales, contributing to the overall complexity of the material. Figure 9b illustrates a nonconstant relationship between the generalized dimensions *Dq* and the moments *q*, indicating high multifractality. This suggests that different regions of the nanocomposites exhibit varying degrees of fractal complexity. The mass exponent *τ(q)* versus *q* curve analysis (Figure 9a) confirms the multifractal nature of the nanocomposites. The nonlinear behavior of this curve underscores nontrivial variations in mass distribution across scales, adding an extra layer to understanding structural complexity. The fractal dimension difference (Δ*f* = *f*(*α_max_*) − *f*(*α_min_*)) was found to be 1.985 and emphasizes the disparity in fractal dimensions in different parts of the coating, highlighting marked variations in structural complexity. Notably, the low roughness of the nanocomposites promoted the formation of a microtexture with unique vertical growth dynamics marked by a strongly multifractal behavior. Comprehending the multifractality mechanism of the nanocomposites offers valuable insights for the design of advanced materials, bioengineering, and other fields where 3D surface architecture plays a pivotal role. Hence, these findings bear substantial implications, especially in the nanomaterial utilization in biomedical applications which demand meticulous control over structural properties, particularly in relation to their surface characteristics. Furthermore, the conjunction of multifractal behavior with microtextural properties, such as low percolation and high topographic uniformity in the nanocomposites, indicates that surface characteristics, including adhesion, friction, and resistance, are governed by distinctive and advantageous surface dynamics. This feature is assigned as beneficial for its application in the biological field.

### 3.6. Fourier Transform Infrared Spectroscopy

**Figure 10:**
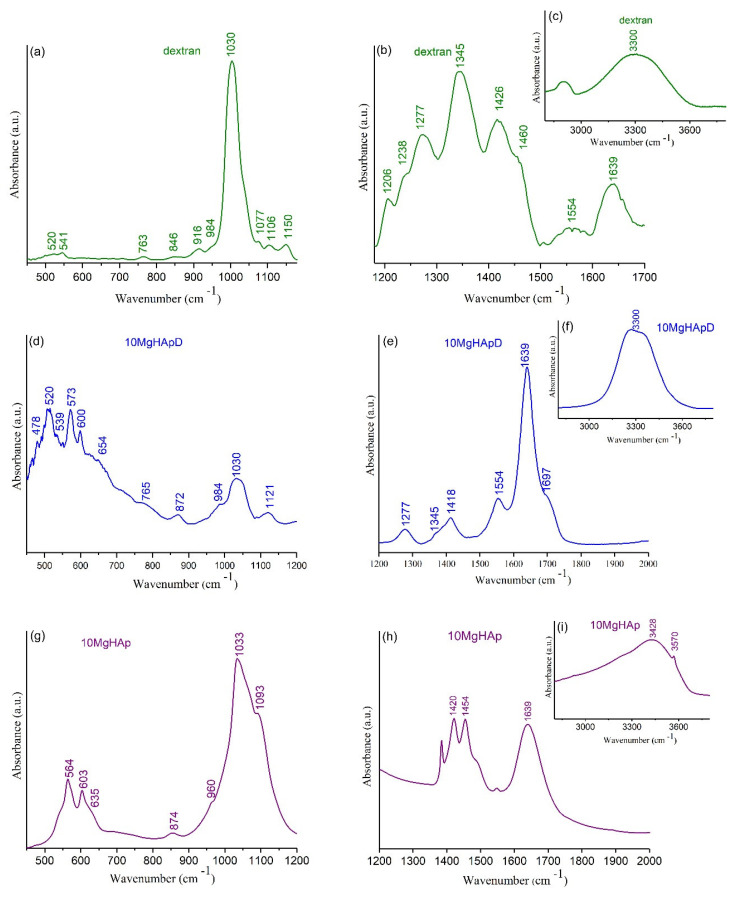
Comparative FTIR-ATR spectra of the dextran powder (**a**–**c**), 10MgHApD nanocomposites (**d**–**f**) and 10MgHAp (**g**–**i**).

The FTIR-ATR spectra of 10MgHApD nanocomposites, magnesium-doped hydroxyapatite (10MgHAp) and dextran powder as reference spectrum are presented comparatively in Figure 10a–i. According to previous studies [33,60], the vibration bands distinctive to HAp were observed together with the characteristic bands of dextran (Figure 10).

For the magnesium-doped hydroxyapatite (10MgHAp) sample, the results of the FTIR studies are presented in Figure 10g–i. The dominant maxima observed in the FTIR spectra could be attributed mainly to the presence of the characteristic vibration of phosphate, hydroxyl groups (from HAp structure) and adsorbed water molecules. The maxima found between 500 and 620 cm^−1^ are characteristic of the ν_4_ triply degenerated asymmetric stretching of phosphate groups [33]. Also, the maxima observed at 960 cm^−1^ are specific to ν_1_ symmetric stretching of the phosphate group. The triply degenerated asymmetric stretching ν_3_ maxima were observed between 980 and 1100 cm^−1^ spectral domain [33]. Moreover, the liberation and stretching vibration of hydroxyl groups are easily observed at 635 cm^−1^ and at around 3570 cm^−1^ [33]. In the FTIR spectra, we could also notice a maximum at around 1420 cm^−1^ and at 1454 cm^−1^, which is usually attributed to the vibration of CO_3_^2−^ groups [33]. The presence of adsorbed water molecules in the studied sample is confirmed by the maxima located at around 1630 cm^−1^ (specific to the bending vibrations) and by the one observed at around 3428 cm^−1^ (specific to the stretching vibrations) of water molecules [33]. The presence of these intense and wide vibration bands confirms that the 10MgHAp samples are strongly hydrated. 

The peak identified at about 465–470 cm^−1^ in FTIR spectra of 10MgHApD (Figure 10d) was assigned to characteristic stretching modes of O-H bands [61]. The bands at about 520–573 and 600 cm^−1^ (Figure 10d) were associated with ν_4_ symmetric P-O stretching vibration of the PO_4_^3−^ group [61,62]. Moreover, the peaks at 765, 846–872 and 984 cm^−1^ identified in Figure 10a,d represent the characteristic band sorption of dextran [63]. The formation of HAp (Figure 10d) is given by the observation of the broad band centered at 960–1121 cm^−1^ assigned to P-O asymmetric stretching of PO_4_^3−^ [61,64]. On the other hand, the peaks that can be observed in 10MgHApD and dextran spectra in the range 1400–1426 cm^−1^ may be assigned to the dextran molecule ν(C–H) and δ(C–H) vibrational modes [63]. In agreement with recent studies [61,63], the presence of peaks at around 872 cm^−1^ (ν_2_ bending vibrations) and 1418–1554 cm^−1^ (ν_3_ asymmetric stretching vibrations) in the FTIR spectra of 10MgHApD was due to CO_3_^2−^ groups (Figure 10e). The stronger peaks observed in the range of 846–1077 cm^−1^ in the FTIR spectra of dextran (Figure 10a) were also identified in the FTIR spectra of 10MgHApD and could be assigned to the stretching vibration of C–O–C [65]. Moreover, the bands in the range 1418–1460 cm^−1^ that can be assigned to C–O–H deformation vibration with contributions of O–C–O symmetric stretching vibration of the carboxylate group [65,66] were present in the FTIR spectra of the two analyzed samples (Figure 10b,e). It can be seen that both analyzed samples (10MgHApD and dextran) were strongly hydrated, as revealed by the intense bands at around 1630–1640 cm^−1^ appertaining to the bending vibrations of adsorbed water molecules (Figure 10b,e). The sample hydration is also confirmed and the intense vibration band is observed at around 3300 cm^−1^ that belongs to the stretching vibrations of water molecules (Figure 10c,f) [33]. Moreover, the results of the FTIR studies indicate that the presence of dextran in the sample induces a broadening and a slight displacement of the maxima associated with the functional groups. Therefore, from this point of view, the features revealed by FTIR measurements are in agreement with those provided by XRD studies.

### 3.7. Antimicrobial Assay

**Figure 11:**
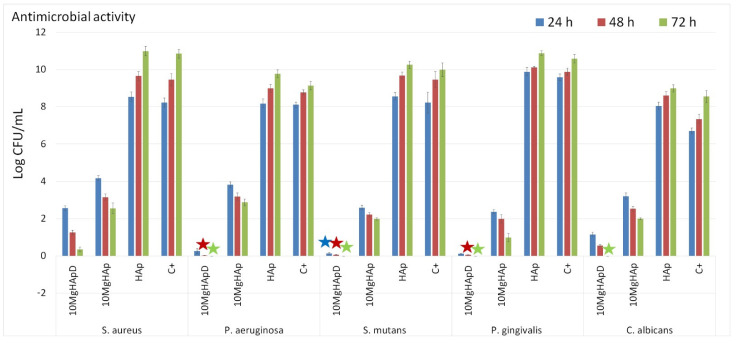
Antimicrobial assay of 10MgHApD nanocomposites, HAp and 10MgHAp nanoparticles against *S. aureus, P. aeruginosa, S. mutans, P. gingivalis* and *C. albicans* microbial strains. The results were considered statistically significant at * *p* < 0.05.

Nowadays, approximately two-thirds of the global population suffer from various dental affections, most encountered being tooth decay, which often leads to the apparition of lesions with various degrees of severity. Even though the surface decay could be easily treated, the tooth could become rapidly unhealthy due to inflammation or infection [67,68,69]. Recently, due to the emergence of microorganisms resistant to conventional therapies, studies regarding the antimicrobial effects of various types of possible antimicrobial agents, such as metallic ions (copper, iron, silver, magnesium, zinc), inorganic nanoparticles and natural polymers, were the focus of researchers due to the need of finding novel solutions having a wide-range action against common pathogens [70,71,72,73,74]. In this context, our study is focused on the development of novel biomaterials based on magnesium-doped hydroxyapatite in dextran matrix for biomedical and dental applications. Therefore, we have studied the antimicrobial activity of the 10MgHApD nanocomposites using *Staphylococcus aureus* ATCC 25923, *Pseudomonas aeruginosa* ATCC 27853, *Streptococcus mutans* ATCC 25175, *Porphyromonas gingivalis* ATCC 33277 and *Candida albicans* ATCC 10231 microbial strains. The in vitro antimicrobial assays were performed in triplicate and the results were presented graphically as mean ± SD. The results of the in vitro antimicrobial assays are depicted in Figure 11. The data suggested that the 10MgHApD nanocomposites exhibited strong inhibitory activity against all the tested microbial strains. In addition, the results of the in vitro antimicrobial experiments showed that the HAp nanoparticles promoted microbial cell development and proliferation. The results highlighted that the microbial cells CFU’s values were higher even than for the control (C+) for all the tested microorganisms for all three incubation times (24, 48 and 72 h). Furthermore, the results also demonstrated that the 10MgHAp nanoparticles exhibited good antimicrobial activity against all the tested microorganisms for all the incubation time intervals. The data also emphasized that the incubation time played an important role in the antimicrobial activity of both 10MgHApD nanocomposites and 10MgHAp nanoparticles. The results showed that the 10MgHApD nanocomposites exhibited bactericidal activity against *P. aeruginosa, S. mutans* and *P. gingivalis* bacterial cells, as well as fungicidal activity against *C. albicans* fungal cells. The data also suggested that the bactericidal and fungicidal effects appear after 48 and 72 h of incubation, respectively. In addition, the results also highlighted that the samples were highly effective in reducing the CFU in the case of dental infection-related bacterial strains, *S. mutans and P. gingivalis,* thus revealing that these types of biocomposites could be successfully used in the development of novel application in dentistry. These results are in good agreement with previously reported data regarding the antimicrobial properties of hydroxyapatite doped with magnesium ions and also of composites based on doped hydroxyapatite in dextran matrix [47,75,76]. Moreover, the data reported by Salem et al. [67] suggested that there are tremendous benefits to employing materials based on magnesium ions in dental restorative applications. 

In their study, Salem et al. [68] showed that the presence of Mg^2+^ promoted an increase in the attachment rate, proliferation, differentiation, alkaline phosphatase activity, and mineralization, leading to a potential improvement of a pulp-capping material. These results, as well as the results obtained regarding the antimicrobial activity of 10MgHApD nanocomposites, suggest that magnesium-based composites might be employed in the development of novel future dental pulp-capping materials that could be used in regenerative endodontics applications. Magnesium is well known as a vital element that plays an important role in the physiological processes within the human body, from supporting muscle and nerve function to regulating blood pressure and contributing to bone health. Even though the exact mechanism is not yet fully understood, in recent years, scientific interest has expanded beyond magnesium’s traditional roles, uncovering unexpected antimicrobial properties. The exact mechanism as to how exactly the magnesium ions exhibit antimicrobial activity still remains unclear, yet several suggestions have been explored in the scientific community. The antimicrobial activity was observed for the first time in early 1900 by Professor Pierre Delbet [77], who found out after numerous tests that a MgCl_2_ solution was the most effective due to the fact that it was not toxic to the surrounding tissue, and it highly increased the leucocyte activity and phagocytosis. Later, Delbet [77], reported that the solution based on MgCl_2_ proved to be efficient in the treatment of various diseases, including the ones related to various microorganisms. In recent years, studies have indicated that antibiotic efficacy could be notably enhanced in the presence of Mg^2+^ ions [78,79]. A prevailing hypothesis suggests that these divalent ions exert an influence on the membranes of bacterial cells. One of the primary proposed mechanisms by which magnesium ions demonstrate antimicrobial effects is through their capacity to interfere with microbial cellular functions. Due to the fact that it is a divalent cation, magnesium has the ability to compete with other metal ions, such as calcium and iron, for binding sites within microbial cells. This competition can disrupt crucial cellular processes, ultimately leading to compromising the growth and survival of bacteria and other microorganisms. In their study, Som et al., regarding “divalent metal ion-triggered activity of a synthetic antimicrobial in cardiolipin membranes” [80], reported that the divalent nature of the cation affected the curvature of the bacterial membrane, which left the bacteria more vulnerable, leading to an increase of the antibiotic’s effects. In addition, Xie et al. [B5] also reported that the antimicrobial effects could be attributed to the fact that magnesium cations help permeabilize the membranes. Magnesium was also reported to be effective in the case of microorganism biofilms by destabilizing and disrupting them. Even though the exact mechanism as to how exactly the magnesium ions have the ability to delay the biofilm formation still remains unclear, several suggestions have been reported [80,81,82,83]. The studies suggested that magnesium ions possess the capability to interact directly with the cell membrane, potentially hindering the formation of biofilm. Another possibility could be attributed to their direct or indirect impact on the regulation of biofilm formation, leading to a delay in the process. Furthermore, a recent investigation illustrated the impact of Mg^2+^ ions against the development of Bacillus biofilm, highlighting a down-regulation of the expression of extracellular matrix genes by more than tenfold [84]. One other mechanism regarding the antimicrobial activity of magnesium composites is attributed to the presence of the existence of high concentrations of OH^−^ on their surface, which leads to an increase of the O^2−^ concentration, causing the destruction of the bacterial cell wall [85]. On the other hand, recently, dextran has gained attention in the scientific community for its diverse applications, particularly in the field of antimicrobial applications. One of the primary proposed antimicrobial mechanisms of dextran was reported to be the ability to inhibit biofilm formation. Dextran has been reported to be able to interfere with the initial stages of biofilm formation by preventing microbial adhesion to the surfaces. This behavior has been attributed to its hydrophilic nature and ability to modify surface properties. Furthermore, it has been reported that dextran’s antimicrobial effects could be related to its ability to disrupt microbial cell membranes. In their studies, Amiri et al. [86] suggested that the polymer interacts with the bacterial membranes, causing destabilization and also an increased permeability. The mechanism responsible for this interaction is not yet fully elucidated, but it is believed that dextran’s physical properties, such as molecular size and charge, could have an important role in compromising the integrity of microbial membranes. This disruption can lead to the leakage of cellular components, ultimately causing the bacterial cell’s death. In addition to the direct effects of magnesium and dextran on microorganisms, both magnesium ions as well as dextran have been found to modulate the host immune response, enhancing the body’s natural defense mechanisms. Dextran could stimulate the production of certain immune mediators, such as cytokines, and promote phagocytosis by immune cells [87,88].

Even though more complex biological studies should be performed to ensure the biological effects of magnesium and dextran on the dentin formation as well as the composite’s antimicrobial activity and cytotoxic dosage, these preliminary results bring significant insight into the exquisite properties of these materials and their potential in future biomedical as well as dentistry applications.

### 3.8. In Vitro Biocompatibility Assay

**Figure 12:**
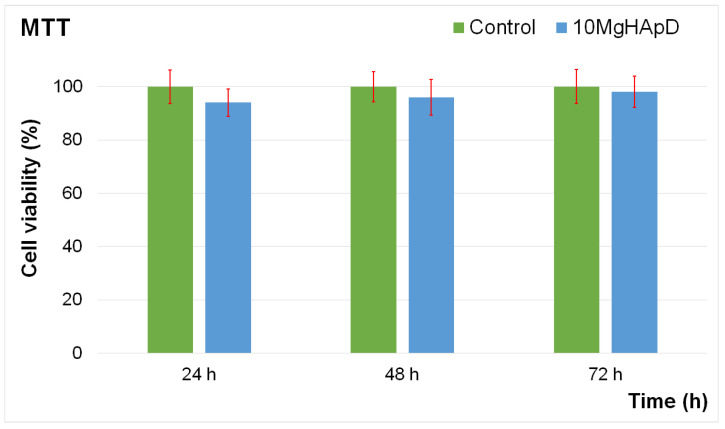
The graphical representation of the cell viability of HGF-1 cells exposed to 10MgHApD nanocomposites for 24, 48 and 72 h. The data are presented as mean ± standard deviation (SD) and are quantified as percentages of control (100% viability). The statistical analysis was performed using the ANOVA single-factor test and *p* ≤ 0.05 was accepted as statistically significant.

Complementary information about the biological properties of the 10MgHApD nanocomposites was acquired by studying the biocompatibility of the nanocomposites using a Human Gingival Fibroblasts (HGF-1) cell line, which exhibits a typical fibroblast morphology. This particular cell line was isolated for the first time in 1989 from the gingiva of a 28-year-old white male patient. The toxicity of the 10MgHApD nanocomposites was evaluated by determining the cell viability of the HGF-1 cells after being exposed to the nanocomposites for three different time intervals. The results of the cell viability assays are depicted as a graphical representation in Figure 12.

The results of the MTT assay emphasized that the nanocomposites did not exhibit any significant toxicity against the HGF-1 cells for any of the incubation times when the data were acquired. The results showed that the viability was not significantly altered compared to the control after 24, 48 and 72 h of exposure with the 10MgHApD nanocomposites, which indicates a good biocompatibility of the samples. The results of the MTT reduction assay represented in Figure 12 showed that the cell viability of the HGF-1 cells exhibited values above 92% after the first 24 h of exposure to the 10MgHApD nanocomposites. Moreover, the findings also determined that the cell viability increased, reaching 96% and 98%, respectively, after 48 h and 72 h of exposure. These findings are in good agreement with other reported data regarding the biological properties of nanocomposites based on HAp, magnesium ions, and different biopolymers [47,89,90,91,92].

## 4. Conclusions

The scanning electronic microscopy (SEM) with energy-dispersive X-ray (EDS) methods in combination with X-ray diffraction (XRD) and Fourier transform infrared spectroscopy technique were applied in this study for the complex investigation of the structure and homogeneity of synthesized 10MgHApD sample. SEM analysis of 10MgHApD nanocomposites presented agglomerated particles with spherical morphology. The qualitative powder-XRD study revealed the nature of the hexagonal HAp. FTIR investigations demonstrated the presence of dextran in the 10MgHApD nanocomposites. Minkowski Functionals indicated nonconventional yet high-quality surface patterns. These findings deepen our understanding of biological surface interactions and have potential implications in materials and biomedicine, which can be further explored for practical applications. The in vitro antimicrobial assay of 10MgHApD nanocomposites against *S. aureus, P. aeruginosa, S. mutans, P. gingivalis* and *C. albicans* microbial strains emphasized that the samples exhibited a strong inhibitory effect on all the tested microbial cells. Moreover, the results also suggested that the 10MgHApD nanocomposite antimicrobial properties were influenced by both the incubation time and also the bacterial cells. In addition, the results demonstrated that 10MgHApD exhibited bactericidal activity against *P. aeruginosa, S. mutans, P. gingivalis* and *C. albicans* microbial strains. The in vitro cell viability assay also demonstrated that 10MgHApD exhibited good biocompatibility properties towards HGF-1 cells. The results obtained showed that this type of nanocomposite based on magnesium-doped hydroxyapatite in dextran matrix could be an effective antimicrobial agent that can be employed for the treatment of various oral diseases as well as dental caries. As a result, it is obviously seen that 10MgHApD nanocomposites have optimal properties for various dental field applications and they can probably be used in other medical or food applications.

## Figures and Tables

**Table 1:** Height parameters of 10MgHApD sample.

Sample	Parameters					
	*Sa* (nm)	*Ssk*	*Sku*	*Sp* (nm)	*Sv* (nm)	*Sz* (nm)
10MgHApD	13.0 ± 0.2	−0.7 ± 0.1	3.1 ± 0.3	68.2 ± 4.7	102.8 ± 0.8	180.9 ± 15.0

**Table 2:** Measures of monofractal parameters of the surface of 10MgHApD nanocomposites.

Sample	Parameters			
10MgHApD	*FD*	*H*	*FS*	*E*
	2.243 ± 0.007	0.757 ± 0.007	0.361 ± 0.160	0.897 ± 0.006

**Table 3:** Multifractal parameters computed from the multifractal spectra of 10MgHApD nanocomposites.

Sample	Parameters					
	α_max_	α_min_	Δ*a*	*f*(*α_max_*)	*f*(*α_min_*)	Δ*f*
10MgHApD	3.109	2.031	1.078	−0.310	1.675	1.985

## Data Availability

Data are available on reasonable demand from the corresponding authors.
